# Descriptive analysis of adherence to mindfulness-based group therapies: online versus face-to-face interventions

**DOI:** 10.1192/j.eurpsy.2022.823

**Published:** 2022-09-01

**Authors:** G. Navarro Oliver, T. Castellanos Villaverde, I. Torrea Araiz, E. Vidal Bermejo, A. Hospital Moreno, I. Louzao Rojas, E. Fernández-Jiménez

**Affiliations:** Hospital Universitario La Paz, Department Of Psychiatry, Clinical Psychology And Mental Health, Madrid, Spain

**Keywords:** group psychotherapy, online intervention, Mindfulness, dropout rate

## Abstract

**Introduction:**

The use of technological supports in psychotherapeutic interventions has been widespread in recent years. Since the COVID-19 pandemic, the increase has been greater. The feasibility of online group interventions has been proved in previous studies. Research comparing dropout rates in group interventions with clinical population that include mindfulness training is infrequent.

**Objectives:**

To compare the difference in dropout rates between online and face-to-face mindfulness-based group interventions.

**Methods:**

This study was carried out in a Mental Health Unit in Colmenar Viejo (Madrid, Spain). One hundred thirty-five adult patients with anxiety disorders were included in group interventions (74 face-to-face; 61 online). The group treatments were Acceptance and Commitment Therapy and a Mindfulness-based Emotional Regulation intervention, during 8 weeks, guided by two Clinical Psychology residents. A descriptive analysis of dropout rates (participants attending 3 or fewer sessions out of the total number of participants starting the intervention) was performed.

**Results:**

Of the 135 patients included, 8 did not participate in the interventions (5 face-to-face; 3 online), which represents a 5.93% rejection rate; 6.76% for the face-to-face intervention and 4.92% for the online intervention. Of the remaining sample (127 participants), a total dropout rate of 12.6% was obtained, with 8.69% in the face-to-face intervention versus 17.24% online.

**Conclusions:**

A higher dropout rate was obtained in online interventions compared to face-to-face, with an increase of almost double. Research on specific factors that may interfere with treatment adherence to online group interventions is needed.

**Disclosure:**

No significant relationships.

